# Evaluation of antibiotic self-medication among outpatients of the state university hospital of Port-Au-Prince, Haiti: a cross-sectional study

**DOI:** 10.11604/pamj.2017.28.4.12589

**Published:** 2017-09-04

**Authors:** Kenny Moise, Joseph Junior Bernard, Jean Hugues Henrys

**Affiliations:** 1 Université Notre-Dame d'Haïti, Faculté de Médecine et des Sciences de la Santé 6, Rue Sapotille, Port-au-Prince, Haiti

**Keywords:** Antibiotics, self-medication, education

## Abstract

In Haiti, where all drugs are available over the counter, self-medication with antibiotics appears as a common practice. Inappropriate use of beta-lactams and macrolides is likely to contribute to the development of antimicrobial resistance. This study aimed to (i) assess the extent of self-medication with antibiotics, (ii) explore the contributing factors (age, gender and educational background) and (iii) identify specific antibiotic drug classes used among patients attending the outpatient clinic of the State University Hospital of Port-au-Prince. A cross-sectional survey among 200 outpatients of the State University Hospital of Port-au-Prince was conducted in December 2014. Face-to-face interviews were conducted using a standardized questionnaire. Parents of pediatric patients were allowed to answer to questions on their behalf. Among the study sample, 45.5% practiced self-medication with antibiotics. It was less prevalent among patients with the highest education level (23.1%; OR: 0.89 (0.5-1.75), p = 0.001). Mild symptoms (28.6%) and vaginal itching (44.4%) were the main reasons for self-medication with antibiotics. Self-medication using amoxicillin was reported by 67.0%. Self-medication with antibiotics is a common practice among Haitian patients and is more common among the less educated. Amoxicillin for urinary tract infections is the most commonly used medication. It is crucial to raise awareness on the dangers of the practice in the population and inforce the current law regarding the use of over the counter antibiotics.

## Introduction

Self-medication, which can be defined as the practice of treating one's own symptoms or disease without the advice or prescription from a medical provider other than a pharmacist [1, 2], is a common practice around the world [3], particularly in developing countries [4, 5]. Self-medication with antibiotics is a major contributor to the development of resistance to antibiotics [6, 7]. Prevalence of self-medication is likely to be influenced by the health system organization, including the availability of physicians who are able to prescribe and the health literacy of the population [8]. In Haiti, antibiotic medication has been regulated since 1955, when a law was passed that forbids the sale of antibiotics without medical prescription. Since 1992, the Haitian Ministry of Health provides essential medicines through governmental programs with the help of the Panamerican Health Organization (PAHO). But a parallel market of contraband pharmaceuticals has developed. Thus it is common in Haiti for antibiotics to be sold illegally over the counter (OTC), by street vendors, by "marketing agents," which are itinerant promoters and vendors of pharmaceutical products and in unlicensed pharmacies. This study is the first evaluation to our knowledge to examine self-medication of antibiotics in Haiti. Our goals were to 1) estimate the prevalence of antibiotic use among an outpatient population in Haiti, 2) explore reasons for self-medication vs. seeking prescriptions for antibiotics and 3) determine which antibiotics are most commonly bought without a prescription.

## Methods

**Study setting and population**: We conducted a cross-sectional study in the outpatient clinic of the State University Hospital of Haiti (HUEH) in Port-au-Prince, Haiti. This clinic receives patients from both the local Port-au-Prince area and from elsewhere in the country. Patients were randomly selected among those seen in the clinic during the time of the study and were invited to participate in the study. The patients came to the clinic for general consultation, for issues not necessarily related to antibiotic treatment failure, inefficiency or any kind of specific disease. Those who gave verbal consent were administered a face-to-face brief interview. Parents of pediatric patients seen in the clinic were allowed to answer to the questions on their behalf. The study was approved by the Bioethics Commission of the Scientific Committee of Université Notre-Dame d'Haïti (UNDH).

**Data collection and analysis**: Data were collected using a questionnaire developed among patients in the Emergency Department of Justinien University Hospital (HUJ) in Cap-Haitian, Haiti. The questionnaire consisted of 7 open-ended questions. Questions included (1) demographic data of respondent, including age, educational level and gender; (2) whether the respondent had used antibiotics that were not prescribed in the past 2 weeks to treat themselves for common symptoms, (3) the reasons for using antibiotics that were not prescribed, (4) the specific name of the antibiotic medication that was used and not prescribed, (5) the source of the medications that were not prescribed and (6) the dose and length of use of antibiotics that were not prescribed. Participants were interviewed in Haitian Creole. Epi Info version 7 was used to analyze data. The prevalence of antibiotic use was reported as well as the percentage of each reason and type of medication. We used Mantel-Haenszel chi square statistic to compare use of non-prescribed antibiotics by sociodemographic characteristics (age, gender and education level of patients).

## Results

The study included 200 patients (76 males and 124 females). The mean age of the participants was 33 years, ranging from 12 to 82 years. Most participants had at least a high school education (57.5% had a high school degree and 19.5% attended university), which 8% were illiterate and 30% achieved only an elementary school education.

**Prevalence of self-medication**: Among the study sample, 45.5% reported having used non-prescribed antibiotics for themselves in the past two weeks. Those with less than a university education were significantly more likely to use non-prescribed antibiotics than those with a university education (OR= 3.46 (1.54-7.75), p = 0.001). Differences by gender and age were not statistically significantly different.

**Types, reasons, sources and patterns of non-prescribed antibiotic use**: Amoxicillin was the most commonly used antibiotic (67% of the participants reported amoxicillin use), followed by ampicillin and metronidazole, used respectively by 51.6% and 22% of the participants. The most common reasons for non-prescribed antibiotics use were vaginal itching (44.4%), burning micturition (39.6%) and lower abdominal pain (22%). Most of the patients decided to obtain antibiotics without a prescription rather than seeing their physician because they or their relatives felt their symptoms were too mild (28.6%) or because they didn't have time to see a physician (25.3% of the patients). Most of the patients who self-medicated with antibiotics observed a 1 to 3-day treatment (41.7%), while 28.6% used them during 7 to 9 days and 15.4% during 10 days or more. The mean duration of utilization was 6 days and ranged from 1 to 60 days. Among the 91 participants who reported non-prescribed antibiotic use, 67% obtained antibiotics from pharmacies, 41.8% from street vendors and 27.5% from marketing agents. Approximatively 70% of the self-medicated patients affirmed that they obtained satisfying results of their practice (69.23%).

## Discussion

**Discussion of differences in results**: This study found that the prevalence of antimicrobial self-medication in a sample of outpatients in Port-au-Prince was 45.5%. This is consistent with findings from a study conducted in Ghana [9]. In Haiti, marketing OTC antibiotics is illegal but most patients buy non-prescribed antibiotics in pharmacies. We believe the relatively high prevalence given these laws are due to lack of ability to enforce the laws and low health literacy in the population. In 2017, easy access to OTC antibiotics in Haiti has not changed. Studies have also shown the importance of education level in the phenomenon of antibiotics self-medication. Among Brazilian students in the healthcare field, 86.4% have practiced self-medication but none used antibiotics [10]. In our study, it was noted that the proportion of antibiotics self-medication is significantly lower among patients who reached the university level compared to those who are illiterate or only have an elementary or secondary level of education. It could be explained by the accumulation of knowledge through reading newspapers, magazines or communication media and debates on health-related subjects among university attendants. As for the symptoms, in our study, non-prescribed antibiotics are mostly used to treat burning micturition, lower abdominal pain and vaginal pruritus. Advertisements by street vendors often suggest that antibiotics are particularly used for the treatment of urinary tract infections (UTI) and sexually transmitted diseases (STD). Therefore, when aware of symptoms suggestive of these diseases, consumers usually buy and consume them. This is another consequence of low health literacy among Haitian patients. Our findings revealed that amoxicillin and ampicillin were the most used antibiotics. In Port-au-Prince, beta-lactam antibiotics count among the most advertised antibiotics and are easily found in the street market and through marketing agents in public buses. Often times, advertisements target sexually active people with symptoms suggestive of UTI or STD.

**Strengths and weaknesses of this study**: This is a well-designed evaluation of antibiotics self-medication habits in this cohort and the first assessment of this problem in Haiti. But it is based on self-report information on an illegal practice so patients may not be entirely forthcoming with their use of OTC antibiotics. The small select sample limits generalizability and external validity, as the study was conducted in a single site. Moreover, there is a potential selection bias as the patients presenting to the clinic may have tried an antibiotic before coming in and therefore have a higher incidence of use, or they may be more likely to come in rather than trying to treat themselves.

**Strengths and weaknesses in relation to other studies**: In relation to other studies, the interview of a general patient population in this study is a strength. But its cross-sectional design did not allow studying the association of key variables.

**Unanswered questions and future research**: This study did not evaluate the association of variables such as economic status, transportation access or distance from healthcare facilities with self-medication. Furthermore, an assessment of the consequences of this practice on bacterial ecology and beta-lactam resistance is now to be performed.

## Conclusion

Antibiotics self-medication is a common practice among Haitian patients. Public health authorities should implement health education programs nationwide regarding the use of antibiotics. Such education programs should target elementary and secondary students in priority, to hinder the influence of medicine vendors and "marketing agents" on their irrational use of antibiotics.

### What is known about this topic

- Self-medication is a common practice in developing countries;
- Prevalence of self-medication is likely to be influenced by the education level.

### What this study adds

- Our study suggests that self-medication with antibiotics is a common practice among Haitian patients;
- Beta-lactam antibiotics are widely used to treat suggestive genitourinary infections;
- Low education is a significant determinant of antibiotics self-medication.

## Competing interests

The authors declare no competing interest.

## References

[1] Pouillard Jean (2001). Rapport adopté lors de la session du conseil national de l'ordre des médecins.

[2] Buclin Thierry (2001). L'automédication: pratique banale, motifs complexes..

[3] Sonam Jain, Malvi Reetesh, Purviya Jeetendra Kumar (2011). Concept of self-medication: a review. Int J Pharm Biol Arch..

[4] Bennadi Darshana (2013). Self-medication: a current challenge. J Basic Clin Pharm..

[5] Moses Ocan, Ebuku Ekwaro A, Bwanga Freddie, Akena Dickens, Richard Sennono, Ogwal-Okeng Jasper, Obua Celestino (2015). Household antimicrobial self-medication: a systematic review and meta-analysis of the burden, risk factors and outcomes in developing countries. BMC Public Health.

[6] Morgan Daniel J, Okeke Iruka N, Laxminarayan Ramanan, Perencevich Eli N, Wei-senberg Scott (2011). Non-prescription antimicrobial use worldwide: a systematic review. Lancet Infect Dis..

[7] Global Antibiotic Resistance Partnership (GARP)-India Working Group (2011). Rationalizing anti-biotic use to limit antibiotic resistance in India. Indian J Med Res..

[8] Jean-Louis Montastruc, Bondon-Guitton Emmanuelle, Abadie Delphine, Lacroix Isabelle, Berreni Aurélia, Pugnet Grégory, Durrieu Geneviève, Sailler Laurent, Giroud Jean-Paul, Damase-Michel Christine (2016). Pharmacovigilance, risks and adverse effects of self-medication. Thérapie..

[9] Donkor Eric S, Tetteh-Quarcoo Patience B, Nartey Patrick, Agyeman Isaac O (2012). Self-medication practices with antibiotics among tertiary level students in Accra, Ghana: a cross-sectional study. Int J Environ Res Public Health.

[10] da Silva Marília Garcez Corrêa, Soares Maria Cristina Flores, Muccillo-Baisch Ana Luiza (2012). Self-medication in university students from the city of Rio Grande, Brasil. BMC Public Health.

